# Synthesis, microstructural and optical characterizations of sol-gel grown gadolinium doped cerium oxide ceramics

**DOI:** 10.1039/d4ra01902d

**Published:** 2024-05-13

**Authors:** S. D. Dhruv, Jayant Kolte, Pankaj Solanki, Milind P. Deshpande, Vanaraj Solanki, Jiten Tailor, Naveen Agrawal, V. A. Patel, J. H. Markna, Bharat Kataria, D. K. Dhruv

**Affiliations:** a NatubhaiV. Patel College of Pure and Applied Sciences, The Charutar Vidya Mandal University Vallabh Vidyanagar-388120 Anand Gujarat India shweta@nvpas.edu.in nvnagrl@gmail.com dhananjay.dhruv@cvmu.edu.in; b School of Physics and Materials Science, Thapar Institute of Engineering and Technology Patiala-147004 Punjab India jayantkolte@thapar.edu; c Department of Nanoscience and Advanced Materials, Saurashtra University Rajkot-360005 Gujarat India pankajsolanki672@gmail.com jaysukh28@gmail.com brkataria22@rediffmail.com; d Department of Physics, Sardar Patel University Vallabh Vidyanagar-388120 Anand Gujarat India vishwadeshpande@yahoo.com; e Dr K. C. Patel R & D Centre, Charotar University of Science and Technology Changa 388421 Gujarat India vanarajsolanki.rnd@charusat.ac.in; f Department of Physics, M. B. Patel Science College, Sardar Patel University Anand 388001 Gujarat India tailorjiten4u@gmail.com; g Sophisticated Instrumentation Centre for Applied Research and Testing Vallabh Vidyanagar 388120 Gujarat India vap@sicart.res.in

## Abstract

In this study, through the utilization of the sol–gel combustion tactic, gadolinium (Gd)-doped cerium oxide (CeO_2_), Ce_1−*x*_Gd_*x*_O_2_ (*x* = 0.00, 0.10, 0.20 and 0.30 (GDC)) ceramics were attained. The synthesized GDC ceramics were investigated using X-ray diffraction (XRD) to scrutinize their crystal structures and phase clarities. The obtained GDC ceramics have a single-phase cubic structure and belong to the crystallographic space group *fm*3̄*m* (225). The measurement of the diffraction angle of each reflection and the subsequent smearing of the renowned Bragg's relation provided coarse *d*-interplanar spacings. The stacking fault (SF) values of pure and Gd-doped CeO_2_ ceramics were assessed. To muse the degree of preferred orientation (*σ*) of crystallites along a crystal plane (*h k l*), the texture coefficient (*C*_*i*_) of each XRD peak of GDC ceramics is gauged. By determining the interplanar distance (*d*_*h k l*_), the Bravais theory sheds light on the material's development. By exploiting Miller indices for the prime (1 1 1) plane, the lattice constants of GDC ceramics and cell volumes were obtained. Multiple techniques were employed to ascertain the microstructural parameters of GDC ceramics. A pyrometer substantiated the density of GDC ceramics. The room temperature (RT) Fourier transform infrared (FTIR) spectra of both un-doped and Gd-doped CeO_2_ were obtained. The UV-vis-NIR spectrometer recorded the GDC ceramics' reflectance (*R*) spectra at RT. For both undoped and Gd-doped CeO_2_, the absorption coefficient (*α*) spectra showed two distinct peaks. The *R*-dependent refractive index (*η*) and the *α*-dependent extinction coefficient (*k*) were determined for all GDC samples. The optical band gap (*E*_g_) was obtained by integrating the Tauc and Kubelka–Munk approaches for GDC ceramics. For each GDC sample, the imaginary (*ε*_*i*_) and real (*ε*_r_) dielectric constants, as well as the dissipation factor (tan *δ*), were determined local to the characteristic wavelength (*λ*_c_). Calculations were made for the Urbach energy (*E*_U_) and Urbach absorption coefficient (*α*_0_) for GDC ceramics. The minimum and maximum values of optical (*σ*_o_) and electrical (*σ*_e_) conductivity for GDC ceramics were determined. The volume (VELF) and surface (SELF) energy loss functions, which depend on the constants *ε*_*i*_ and *ε*_r_, were used to measure electrons' energy loss rates as they travel across the surface. Raman spectroscopy revealed various vibrational modes in GDC ceramics. Finally, the implications are discussed herein.

## Abstract 2

In this study, through the utilization of the sol–gel combustion tactic, gadolinium (Gd)-doped cerium oxide (CeO_2_), Ce_1−*x*_Gd_*x*_O_2_ (*x* = 0.00, 0.10, 0.20 and 0.30 (GDC)) ceramics were attained.

## Graphical abstract

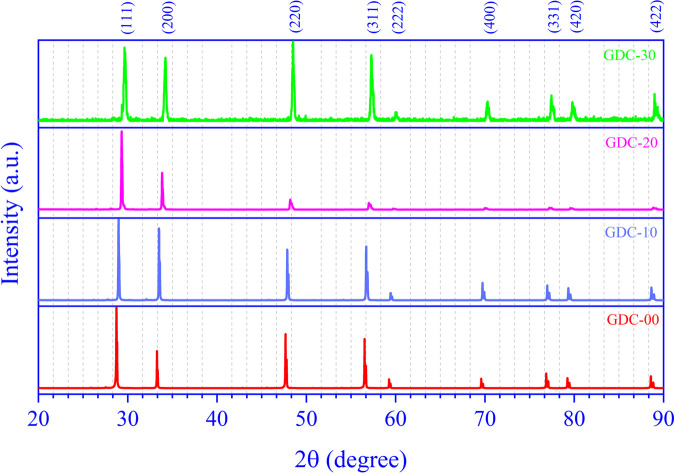

## 1. Introduction

Several researchers have studied cerium oxide (CeO_2_), and their verdicts cover an all-embracing gamut of potential uses. The rare earth oxide CeO_2_ has a lot of latent applications in electro-optical devices,^1^ optoelectronic devices,^2^ and microelectronic devices.^3^ Therefore, it is indispensable to fine-tune the morphological and structural features of CeO_2_ for diverse applications, such as photocatalysis,^4^ gas sensors,^5^ solar cells,^6^ fuel cells,^7^ polishing materials,^8^ corrosion protection coating for all metals and alloys,^9^ materials for superconductor electrodes in electrochemistry, and antibacterial medication.

The cubic fluorite structure of cerium oxide is retained up to its melting point (≃2700 K) and is chemically inactive toward most electrode materials. The ionic conductivity of CeO_2_ is meager, making it inapt for fuel cell applications because of its low oxygen vacancy concentration. The conductivity can be enhanced by including aliovalent dopants such as Sm, Nd, Gd, Ca, Cu, and Pr in the cerium oxide.^10^ Gadolinium (Gd^3+^: 119.3 pm), whose ionic radius nearly resembles that of cerium oxide (Ce^4+^; 111 pm), is the most often used dopant in cerium oxide.^11^ Oxygen vacancies are created^12^ due to Gd^3+^ substitution in the cerium oxide lattice, which increases the electrolyte's ionic conductivity. Gadolinium-doped cerium oxide has a more excellent ionic conductivity than conventional yttria-stabilized zirconia at intermediate temperatures (775–975 K), making it a suitable replacement in solid oxide fuel cells in this temperature range. The temperature, defect dissociation, size of the dopant, the microstructure of sintered pellets, oxygen partial pressure, degree of doping, and sample preparation technique all hinder the ionic conductivity of gadolinium-doped cerium oxide.^13^ Highly dense ceramics must be synthesised because ionic conductivity is influenced by the synthesis process and the ceramic's density. It is crucial to reduce the sintering temperature because ceramics composed of cerium oxide require high sintering temperatures (1875 K), which lengthen fabrication times and raise the cost of these ceramics. This temperature can be reduced by employing initial nano-sized powders.

As a result of its inherent limitations, no crystal can be considered flawless; an ideal crystal would appear to extend infinitely in every direction. Changes from highly crystallinity-preserving materials cause diffraction peak broadening. Crystallite size (*D*) and strain (*ε*) are the main metrics derived from XRD peak width analysis. Several theoretical methods can be used to determine the average *D* and *ε*. These include the Debye Scherrer (D–S) method, Williamson–Hall (W–H) method with uniform deformation models, Size–Strain plot (SSP), and Halder–Wagner (H–W) method.

One of the most fascinating and advantageous features of semiconductors is their optical characteristics. Dispersion phenomena cause electromagnetic waves to lose energy as they travel, which makes the real component, called the refractive index (*η*), and the imaginary part, called the extinction coefficient (*k*), more complicated. It has been noted that the most precise ways to determine the energy gap (*E*_g_) are by optical absorption (*A*) measurements.^14^

Using the sol–gel combustion process, this paper describes the production of gadolinium (Gd)-doped cerium oxide (CeO_2_) and gives detailed information about the microstructural and optical properties of intrinsic and Gd-doped CeO_2_ ceramics. It is anticipated that researchers worldwide will leverage results to sneak them into all sorts of devices.

## 2. Materials and methods

### 2.1. Synthesis

The 4N untainted cerium(iii) nitrate hexahydrate [Ce(NO_3_)_3_·6H_2_O] (Merck, Germany) and gadolinium(iii) nitrate hydrate [Gd(NO_3_)_3_·H_2_O] (Merck, Germany) were used as metal precursors to concoct powders of composition Ce_1−*x*_Gd_*x*_O_2−*δ*_ (*x* = 0.00, 0.10, 0.20 and 0.30, denoted as GDC-00, GDC-10, GDC-20, and GDC-30, respectively). 3N untainted ethylene glycol [C_2_H_6_O_2_] (Loba Chemie, India) and citric acid monohydrate [C_6_H_8_O_7_] (Loba Chemie, India) were used for the polymerization process. The metal precursors were dissolved in double distilled water (DDW); the solution was then mixed with ethylene glycol and citric acid. The molar ratios of citric acid: metal oxide and citric acid: ethylene glycol were kept at 1 : 2 and 1 : 4, respectively. After 2.0 hours of sonication at a high frequency (20 kHz) and high power of 550 W with a 15 second on–off cycle, the mixture was subjected to a temperature of 65–75 °C and stirred on a hot plate to expedite gelation. The ensuing dark brown gel was then placed in an oven at 260 °C to activate auto incineration. The resulting ash-like substance was heated to 550 °C for 2.0 hours to eliminate organic excess. The calcinated powder was pressed at 110 MPa hydraulic pressure for pellet (10 mm diameter and 03 mm thickness) creation and sintered at 1500 °C for 6 h.

### 2.2. Characterization

The crystal structures of GDC ceramics were determined by X-ray diffraction (XRD) (model: X'Pert MPD; make: Philips, Holland) exploiting the typical CuKα radiation (≃0.1541 nm) in the 2*θ* range 20° to 90°. The densities of GDC ceramics were measured using a densitometer (model: Smart Pycno 30, make: Smart Instruments Company Pvt. Ltd., India). Fourier transform infrared (FTIR) spectroscopy (model: Spectrum-GX, make: PerkinElmer, USA) was executed at room temperature (RT) (≃303 K) in the wavenumber (*ν̄*) range 4400–300 cm^−1^ to identify the functional groups extant in the synthesized GDC ceramics. A UV-vis-NIR spectrometer (model: Lambda 19, make: PerkinElmer, USA) was used to acquire the optical strictures of GDC ceramics. A micro-Raman apparatus (Model: STR 500 spectrometer) was used for conducting the RT Raman investigation on the GDC ceramics, which entailed stimulation at 532 nm in their vibrational mode.

## 3. Results and discussion

### 3.1. X-ray diffraction (XRD) analysis

**Fig. 1:**
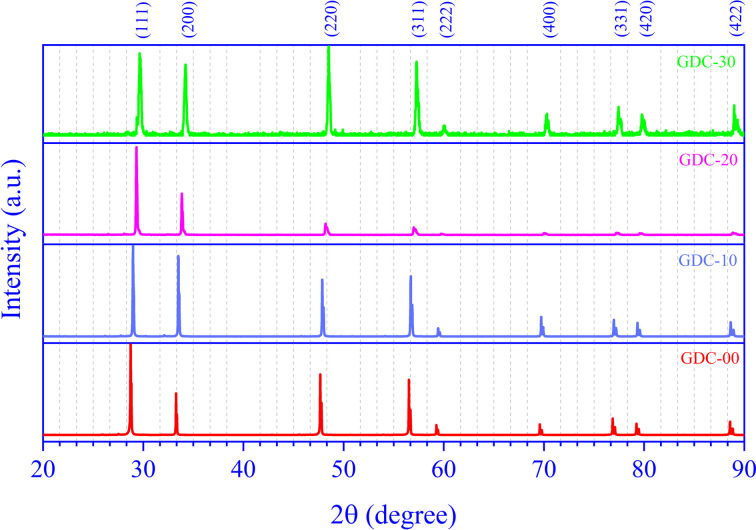
X-ray diffraction patterns of GDC-00, 10, -20, and -30.

RT X-ray diffraction (XRD) revealed the effective synthesis of the polycrystalline GDC compounds. Fig. 1 demonstrates the GDC ceramic systems' powder X-ray diffractograms, which match the standard ICDD data reasonably well (ICDD card numbers 00-043-1002, 00-075-0161, 00-050-0201, and 00-046-0507 for GDC-00, GDC-10, GDC-20, and GDC-30, respectively). There is clear evidence of both pure CeO_2_ and systems doped with Gd from the diffraction peaks that correspond to the reflection planes (1 1 1), (2 2 0), (3 1 1), (2 2 2), (4 0 0), (3 3 1), (4 2 0), and (4 2 2). The strong XRD peak intensities and low full width at half maximum (FWHM) values in the produced GDC ceramic samples indicate high crystallinity. The GDC ceramics have a single-phase cubic structure and fit the crystallographic space group (SG) *fm*3̄*m* (225).

The high-intensity XRD peaks (1 1 1), (2 0 0), (2 2 0), and (3 1 1) were used for a thorough assessment.

The *d*-interplanar spacings were determined by gauging the diffraction angle of each reflection according to the eminent Bragg's relation, eqn (1):^15^1
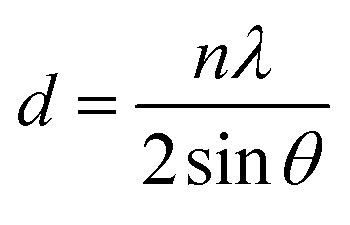


The *d*-spacings for each reflection are given in Table 1.

**Table 1:** The *d*-spacing, SF, texture coefficient, and standard deviation of GDC-00, -10, -20, and -30 ceramics'

System	*h*	*k*	*l*	2*θ* (°)	Intensity	*d* values (nm)				SF	*C*_*i*_	*σ*
						Reported^a^	Observed^b^	Calculated^c^	Calculated^d^			
GDC-00	1	1	1	28.7430	100.00	0.3124	0.3103	0.3104	0.3104	0.001013	0.7501	0.1566
	2	0	0	33.2750	039.80	0.2706	0.2690	0.2691	0.2691	0.000865	1.1057	
	2	2	0	47.6670	060.60	0.1913	0.1906	0.1907	0.1907	0.000805	0.9882	
	3	1	1	56.5240	052.40	0.1632	0.1627	0.1627	0.1627	0.000730	1.1560	
GDC-10	1	1	1	28.9820	100.00	0.3128	0.3078	0.3079	0.3079	0.001052	0.5658	0.4503
	2	0	0	33.5050	086.80	0.2709	0.2672	0.2673	0.2673	0.001056	1.7353	
	2	2	0	47.8690	059.10	0.1916	0.1899	0.1899	0.1899	0.000823	0.7145	
	3	1	1	56.7000	060.20	0.1634	0.1622	0.1623	0.1623	0.000770	0.9844	
GDC-20	1	1	1	29.3250	100.00	0.3126	0.3043	0.3044	0.3044	0.00111	2.3349	0.6401
	2	0	0	33.8530	044.60	0.2708	0.2646	0.2646	0.2646	0.00110	0.7498	
	2	2	0	48.2070	013.40	0.1915	0.1886	0.1887	0.1887	0.00154	0.5928	
	3	1	1	57.0100	008.40	0.1634	0.1614	0.1615	0.1615	0.00167	0.3225	
GDC-30	1	1	1	29.6760	089.40	0.3135	0.3008	0.3009	0.3009	0.00230	0.3998	0.1246
	2	0	0	34.2170	081.90	0.2715	0.2618	0.2619	0.2619	0.00177	1.2630	
	2	2	0	48.5160	100.00	0.1920	0.1875	0.1875	0.1875	0.00128	1.0908	
	3	1	1	57.2890	086.40	0.1638	0.1607	0.1607	0.1607	0.00120	1.2464	

a ICDD card numbers: GDC-00: 00-043-1002, GDC-10: 01-075-0161, GDC-20: 00-050-0201, and GDC-30: 00-046-0507.

b XRD pattern of the bulk.

c Bragg's law.

d Bravais theory.

One crystallographic flaw is the stacking fault (SF), which manifests as planar defects in two dimensions and indicates the incompetence of crystallographic planes. Table 1 shows the SFs of Gd-doped and pure CeO_2_ for four broad peaks, as predicted by eqn (2):^16^2
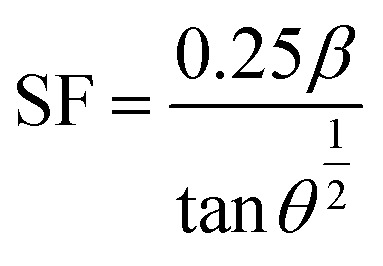
Here, *β* is a measure of the full breadth/width at half maximum of the diffraction peak.

Utilizing eqn (3), one can discern the crystallites' preferred orientation along a crystal plane (*h k l*) by gauging the texture coefficient (*C*_*i*_) of each XRD peak of GDC ceramics:^17^3
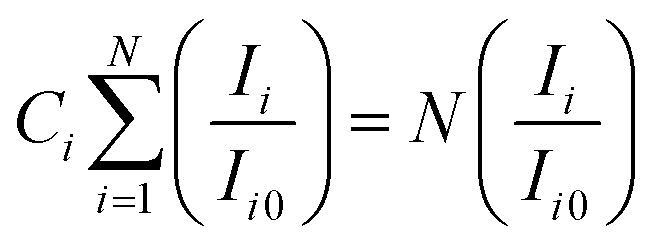


*C*_*i*_ signifies the texture coefficient of the plane *i*, *I*_*i*_ symbolizes the integral intensity, *I*_*i*0_ designates the integral intensity of the reported data of the corresponding peak *i*; *N*, which is equal to 4 in this investigation, represents the number of reflections in the X-ray diffraction pattern being examined. An irrationally oriented sample has a *C*_*i*_ value of one for every reflection; a value greater than one indicates the preferred orientation of the crystallites in that particular direction.

A determination of the GDC ceramics' degree of preferred orientation (*σ*) can be made by calculating the standard deviation of all *C*_*i*_ values using eqn (4):^17^4
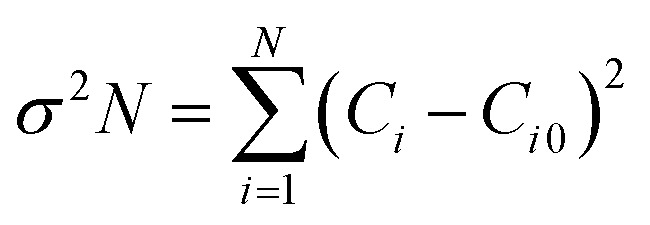


The texture coefficient *C*_*i*0_ is scaled to 1, and *σ* is the gauge of the sample's orientation; a value of zero evokes a random orientation, while a more excellent value of *σ* proves a modified preferred orientation. Table 1 boasts access to the *C*_*i*_ values of the four prominent peaks and the *σ* value for GDC ceramics.

To gain insight into the evolution of the material, the Bravais theory was used to find the distance (*d*_*h k l*_) between the crystal planes. The theory propositions the related distance between crystal planes (*d*_*h k l*_) and the growth rate of the plane (*R*_*h k l*_) as 
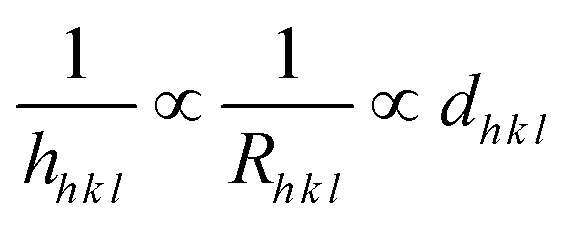
. A collection of lattice parameters (*a*, *b*, *c*, *α*, *β*, *γ*) for the GDC crystal system can be used to compute the *d*_*h k l*_ according to eqn (5):^18^5
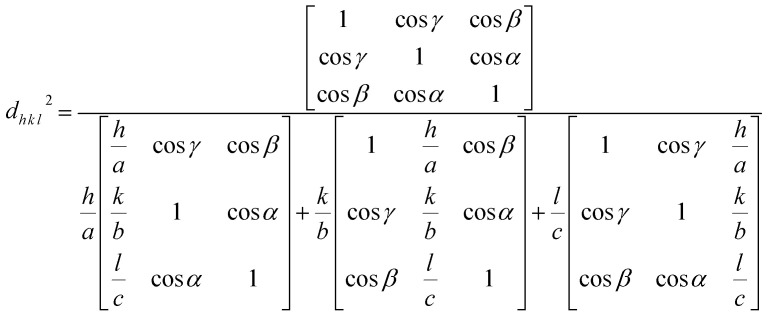


It is possible to deduce the significance of the GDC's (1 1 1) plane from the estimated *d*_*h k l*_, which unveils that *d*_1 1 1_ is the greatest (Table 1), emphasizing that *h*_1 1 1_ is the smallest. This is due to, according to Bravais theory,^18^ the growth rate of the (1 1 1) plane *R*_1 1 1_ being the smallest.

For the cubic crystal system, eqn (6) (ref. 19) shows a relationship between the lattice parameter (*a*) and *d*-value for each Miller index.

6 *a*^2^ = *d*^2^ (*h*^2^ + *k*^2^ + *l*^2^)

### 3.2. Microstructural parameter analysis

It is crucial to research the properties of a compound before using it in contexts of cutting-edge technology. By examining its X-ray diffractogram, one can learn about the microstructure of a laboratory-synthesized compound and extract data on its lattice parameters (*a*, *b*, and *c*), *D*, *δ*, and *ε*.^19,20^ Lattice strain is the internal stress that compresses or expands the length of a unit cell compared to its initial size. This article boasts a range of approaches to evaluating the microscopic structure of GDC.

#### 3.2.1. The Nelson–Riley (N–R) method

The N–R tactic, the error function, can be employed to determine the lattice parameter (*a*) of cubic GDC ceramics. To obtain the error function from eqn (7), the N–R graph was constructed by combining the computed lattice parameters from several planes:^21^7
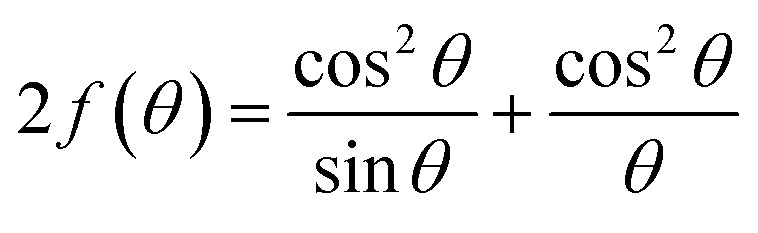


**Fig. 2:**
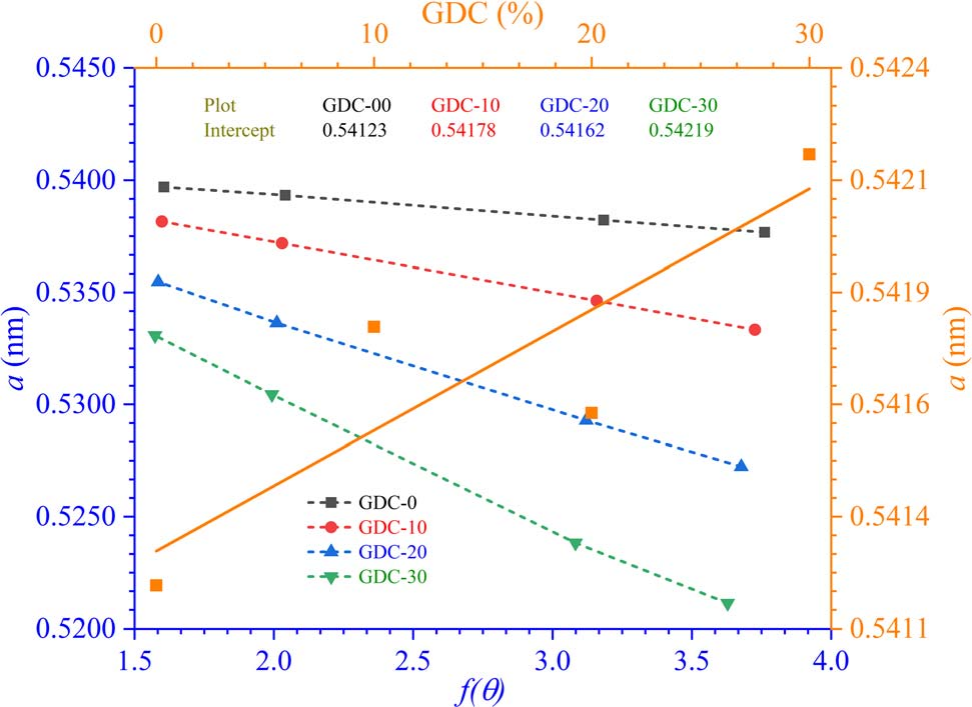
N–R plots for GDC-00, -10, -20, and -30 ceramics.

Table 2 displays the results, and Fig. 2 shows the N–R plot, where the intercept was utilized to determine *a* (where *a* = intercept).

**Table 2:** Lattice parameters and unit cell volume of GDC-00, -10, -20, and -30 ceramics

System	*a* (nm)		*V* (=*a*^3^) (nm^3^)		Reference^b^
	Observed^a^	Reported^b^	Observed^a^	Reported^b^	
GDC-00	0.5412	0.5411	0.1585	0.1584	00-043-1002
GDC-10	0.5418	0.5418	0.1590	0.1590	01-075-0161
GDC-20	0.5416	0.5420	0.1589	0.1592	00-050-0201
GDC-30	0.5422	0.5431	0.1594	0.1602	00-046-0507

a N–R method.

b ICDD card number.

The lattice parameters and volume computed for GDC are displayed in Table 2

Lattice parameter (*a*) values increased with increasing amounts of Gd-doped into the parent CeO_2_, as shown in Fig. 2. The conversion of specific fractions of Ce^4+^ into Ce^3+^, a form of cerium with a greater ionic radius (1.14 Å compared to 0.97 Å for Ce^4+^), could be a likely reason why the lattice parameter value increases when doped.^22^

#### 3.2.2. The Scherrer method

The Scherrer eqn (8) can be used to estimate *D* from the breadth/width of XRD lines:^19^

8 *Dβ* cos *θ* = *Kλ*

Scherrer's constant is denoted as *K* (≃0.9).

On rearranging eqn (8), we obtain eqn (9),9
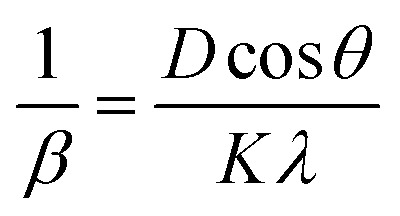


**Fig. 3:**
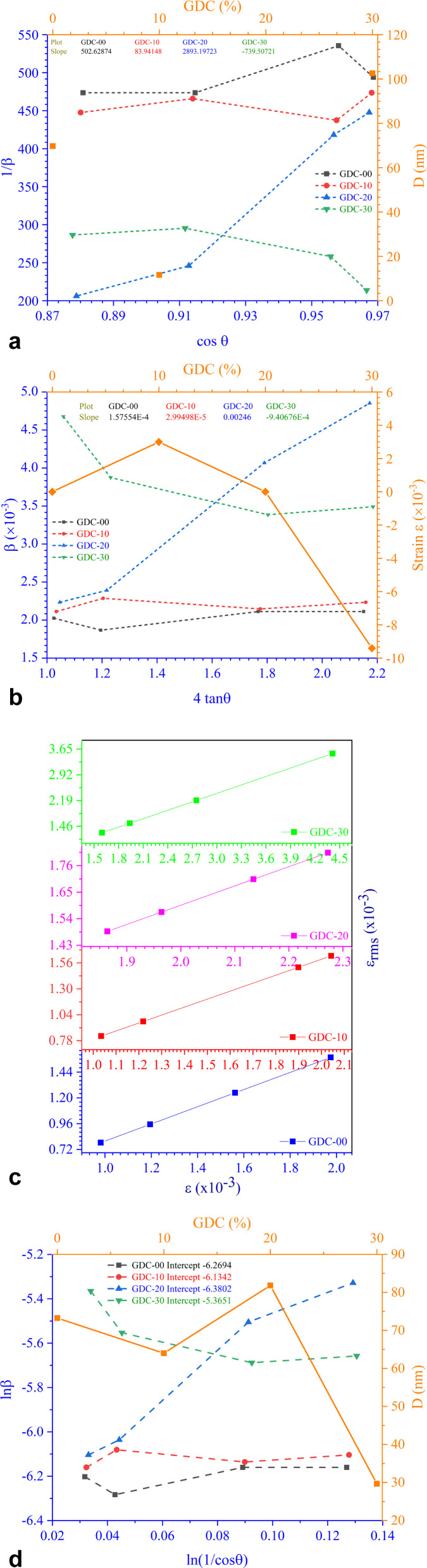
(a) Scherrer, (b) S–W, (c) *ε*_rms_ − *ε*, and (d) Monshi plots for GDC-00, -10, 20, and -30 ceramics.

Fig. 3(a) shows the Scherrer plot, and Table 3 provides the average *D* (=*Kλ*Slope). Table 3 does not include the *D* value for GDC-20 since the current experiment produced a *D* value greater than 200 nm, which is outside the range of validity for Scherrer's computation, which is limited to average *D* values between 100 and 200 nm.^23^ There is a negative correlation between the size of a crystallite and its full width at half maximum (FWHM); therefore, when the parent CeO_2_ is doped with Gd, the FWHM of all planes increases, leading to smaller crystallites.

**Table 3:** Crystallite size and strain values of GDC-00, -10, -20, and -30 ceramics^a^

System	Method	Scherrer	Monshi	W–H (UDM)	SSP	H–W
GDC-00	*D* (nm)	69.7096	73.2469	72.6126	70.7602	78.7724
	*ε* (×10^−3^)	0.0394	—	−0.0170	−0.1641	−1.0646
GDC-10	*D* (nm)	11.6418	63.9861	60.5633	64.8084	71.9114
	*ε* (×10^−3^)	0.0075	—	−0.1734	−0.1809	−1.1738
GDC-20	*D* (nm)	—	81.8293	294.8843	185.3047	205.8943
	*ε* (×10^−3^)	0.6150	—	2.5	1.4885	9.6591
GDC-30	*D* (nm)	102.5623	29.6531	24.0781	28.5370	31.7365
	*ε* (×10^−3^)	0.2352	—	−1.51	−0.8737	−5.6698

a Compressive stress, denoted by a negative *ε* value for GDC, results from the assertion of equal and opposing pressures, which in turn causes the crystalline structure to shrink.^32,33^

##### 3.2.2.1. The Stokes–Wilson (S–W) method

The lattice *ε* predisposed by crystal imperfection and chaos was resolute in the un-doped and Gd-doped CeO_2_ samples using the Stokes–Wilson (S–W) relation eqn (10):^24^10
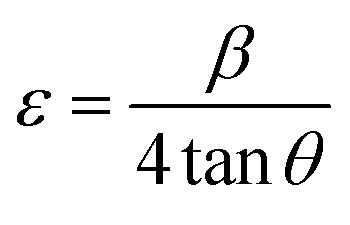


Table 3 shows the average *ε* (=Slope) speculated from the S–W plot (Fig. 3(b)), and it is that disparity in radii between the host cation (Ce) and the dopant (Gd) that stirs strain in the host lattice.

With the aid of the S–W relation eqn (11), it is possible to compute the micro-strain (*ε*_rms_) along each mineralographic plane:^24^

11 *ε*_rms_^2^ = 0.64*ε*^2^

Fig. 3(c) shows a plot of micro-strain (*ε*) *vs.* root mean square strain (*ε*_rms_), demonstrating that the crystallographic direction of the lattice planes is consistent. When plotted against an abscissa, the data points should form an angle of 45 degrees, indicating that *ε* has a linear relationship with *ε*_rms_.^25^

#### 3.2.3. The Monshi method

In their report, Rabiei *et al.*^26^ confirmed that Scherrer's equation increased the projected nanocrystalline sizes for increasing 2*θ* values. An example of how Monshi modified Scherrer's original formula is seen in eqn (12).^27^12
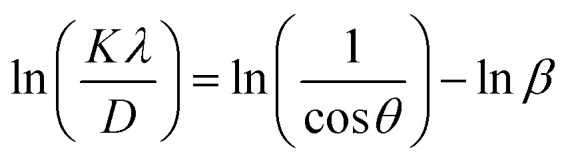


The average *D*, which is equal to *Kλe*^Intercept^−1^^, was figured out in Fig. 3(d) and is accessible in Table 3.

#### 3.2.4. The Williamson–Smallman's (W–S) method

For GDC-00, GDC-10, and GDC-30, the values of *D* were obtained using Scherrer's strategy in eqn (9) and used in eqn (13) for determining the dislocation densities (*δ*) of 0.2058 × 10^−3^, 7.3784 × 10^−3^, and 0.0951 × 10^−3^ lines-nm^−2^, respectively.^19^

13 *δD*^2^ = 1

#### 3.2.5. The Williamson–Hall (W–H) method

Despite considering the effects of *D* on the XRD peak broadening, the Scherrer method fails to explain lattice microstructures or intrinsic *ε*, which remains in powder form because of defects. Different effects on the line width are postulated to result from other *D* and *ε* in the W–H method, which is why line broadening depends on the diffraction angle 2*θ*. Since the broadening of the line can be attributable to either a Lorentzian or a Gaussian function, the values of *D* and *ε* that were obtained are the median values of the line. Uniform deformation models (UDM) can be derived from the modified W–H equation, allowing for the measurement of *D* and microdeformation.^28^

##### 3.2.5.1. The UDM method

Utilizing the W–H approach and eqn (14), one can estimate that *D* and *ε* were affected in large quantities due to crystal defects and distortion.^29^

14 *βD* cos *θ* = 4*εD* sin *θ* + *Kλ*

**Fig. 4:**
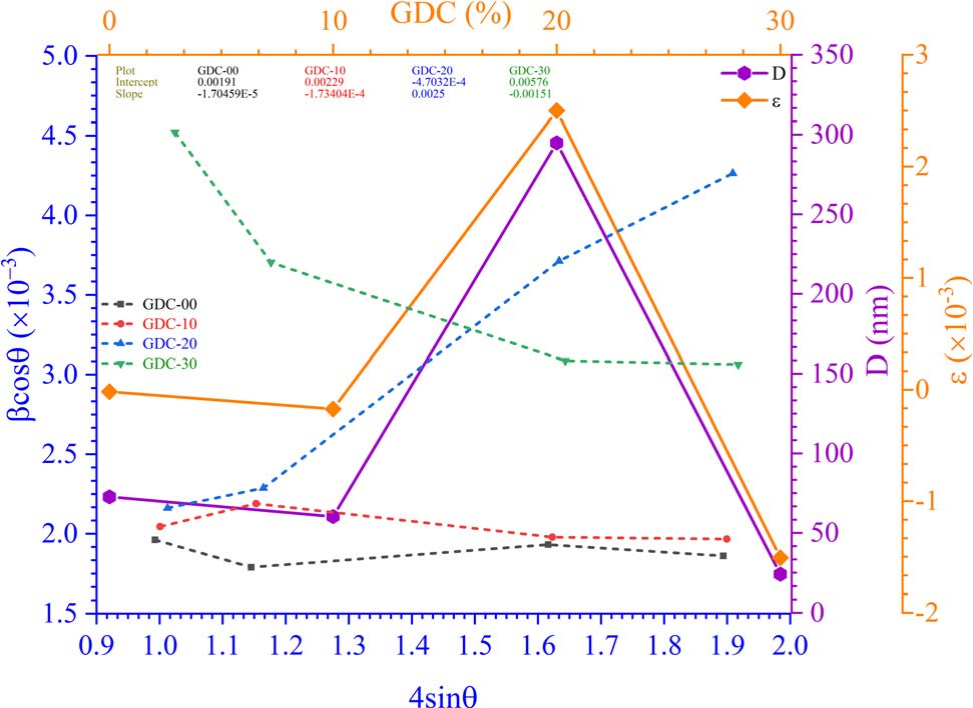
W–H plots (UDM) for GDC-00, -10, -20, and -30 ceramics.

The values of *ε* (=Slope) and *D* (=*Kλ*Intercept^−1^) are shown in Table 3, derived from the straight-line plot in Fig. 4.

#### 3.2.6. The size-strain plot (SSP) method

According to the W–H scrutiny, the diffraction angle 2*θ* portrays peak broadening as an aftermath of the juxtaposed effects of *D* and *ε* -induced broadening.

The SSP approach considers the XRD line analysis a combination of Lorentzian and Gaussian functions and uses eqn (15) to compute *D* and *ε*:^30^15
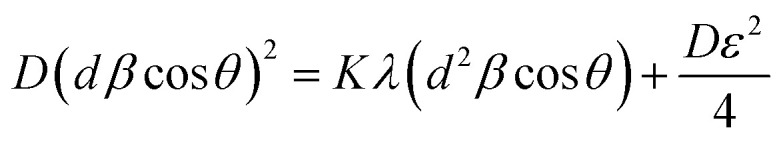


**Fig. 5:**
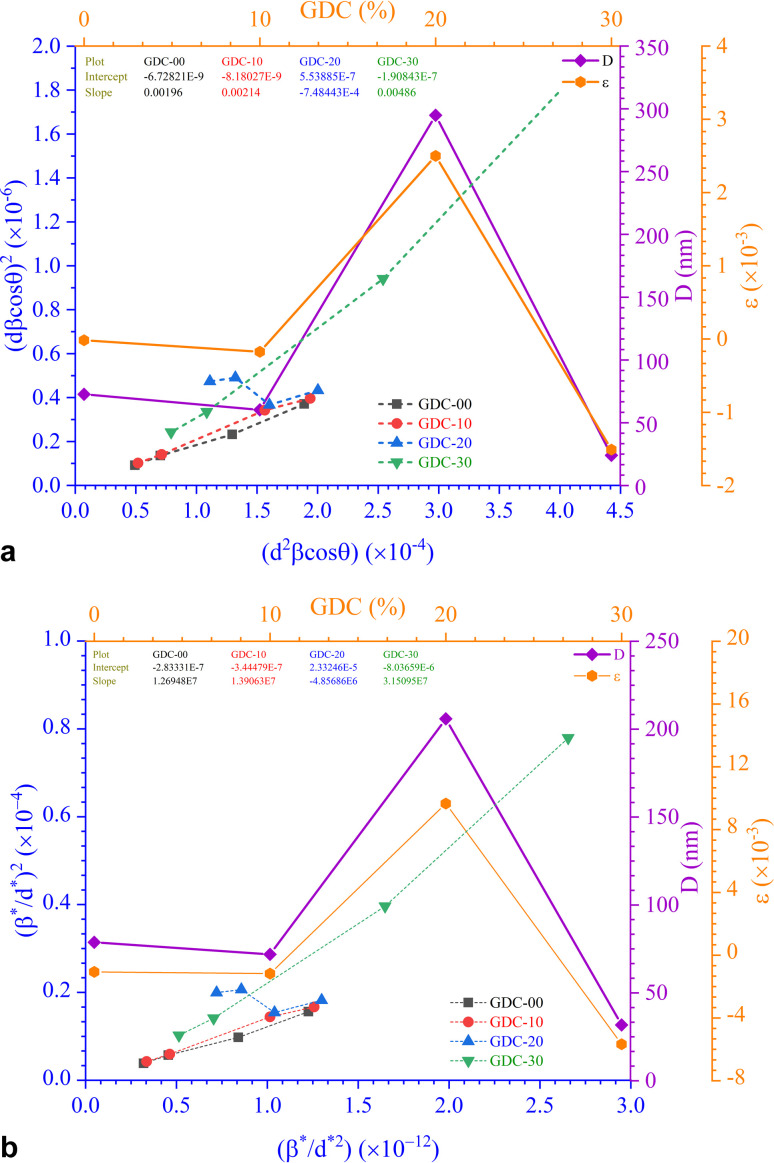
(a) SSP and (b) H–W plots for GDC-00, -10, −20, and −30 ceramics.

D, which is equal to *Kλ*Slope^−1^, and *ε*, which is equal to 
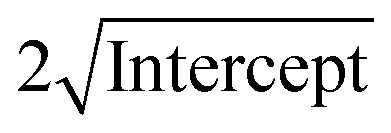
, were obtained from the linear fits for GDC indicated in Fig. 5(a); the results can be found in Table 3.

#### 3.2.7. The Halder–Wagner (H–W) method

The SSP method's XRD peak width assumption is false because neither the mid-peak area nor the XRD peak's tail could be mapped using the corresponding Gaussian nor Lorentzian functions. Using the convolution of Gaussian and Lorentzian functions, we can debunk the H–W analysis's premise that peak broadening is the Voigt function. Eqn (16) and Fig. 5(b) illustrate the connection between *D* and *ε* concerning the H–W technique:^31^16
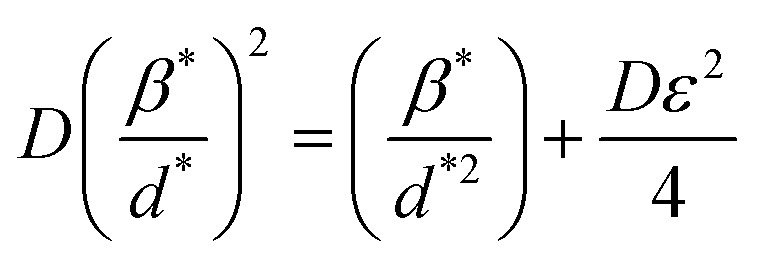
Here, 
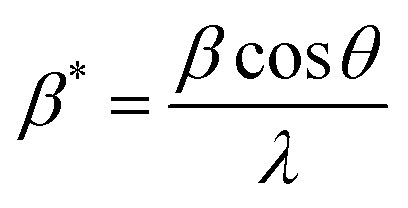
 and 
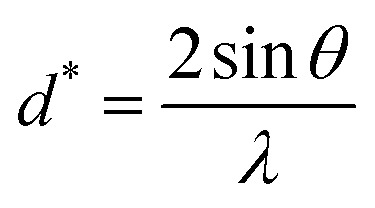


The straight line in plot 5 (b) gives *D*, which is equal to slope^−1^, and *ε* can be determined from the intercept (equal to 
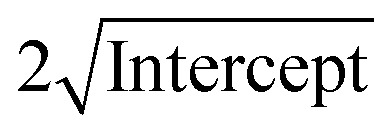
). The results can be seen in Table 3.

### 3.3. Density measurements

Results from densitometer measurements of the density of GDC ceramics designate that the particles are unvarying in size and shape throughout. The standards for GDC-00, -10, -20, and -30 (ICDD card numbers 00-043-1002, 01-075-0161, 00-050-0201, and 00-046-0507, respectively) agree with the study's average density values of 7.2089, 7.1562, 7.2286, and 7.2473 gm cm^−3^.

### 3.4. Fourier transform infrared (FTIR) analysis

**Fig. 6:**
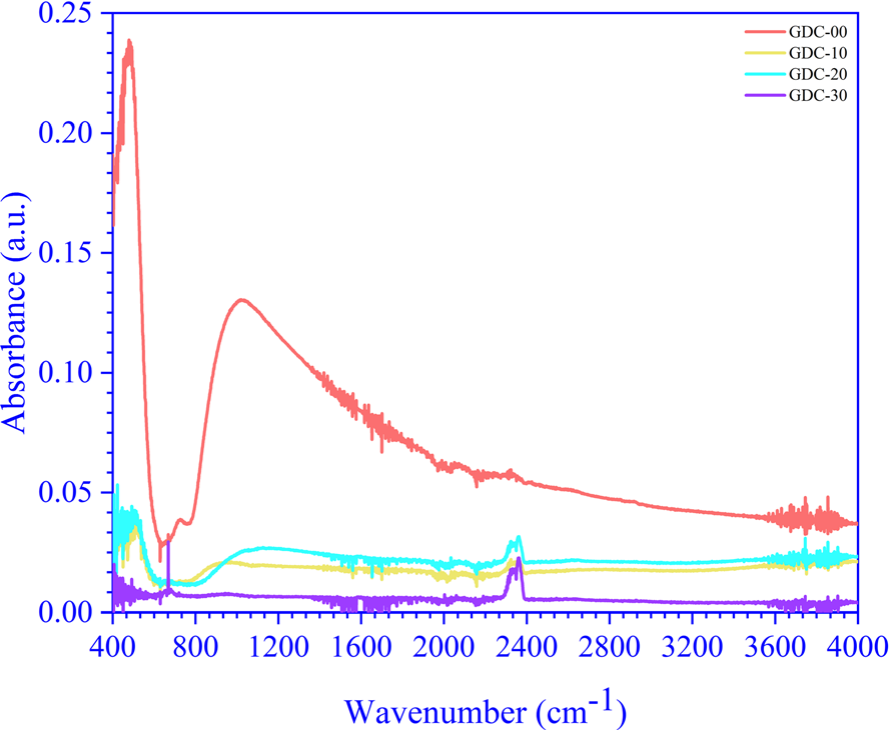
FTIR spectra of GDC-00, -10, -20, and -30 ceramics.

The RT FTIR spectra of un-doped and Gd-doped CeO_2_ recorded in the 4400 to 300 cm^−1^ wavenumber (*ν̄*) range are shown in Fig. 6. The un-doped CeO_2_ (GDC-00) spectrum exhibited a higher absorbance intensity than the Gd-doped spectra (GDC-10, -20, and -30); the peaks below 700 cm^−1^ represent the stretching vibrational modes of Ce–O and Gd-doped Ce–O. The broad peaks at 1025, 897, 1131, and 952 cm^−1^ are due to CeO_2_ (GDC-00), Gd_0.1_Ce_0.9_O_2_ (GDC-10), Gd_0.2_Ce_0.8_O_2_ (GDC-20), and Gd_0.3_Ce_0.7_O_2_ (GDC-30), respectively, corresponding to Ce–O and Gd–O–Ce bonds. The peaks between 1400–1500 cm^−1^ and 2350–2360 cm^−1^ are caused by carbonate-type species associated with oxide particle surfaces, such as ambient CO_2_, interacting with cerium cations to form species that would decompose following sample treatment at high temperatures.^34^ A study conducted by Aboud *et al.*^35^ showed that Gd-doped CeO_2_ gas sensors have improved sensitivity, stability, operating temperature, and the capacity to detect CO_2_ gas. The doping effect of Gd in CeO_2_ is pragmatic and occurs between 2350–2360 cm^−1^. Residual water and hydroxy groups were indicated by a large band between 3600–3800 cm^−1^, agreeing with the O–H stretching vibration and near 1600 cm^−1^ due to the bending vibration of the associated H–O–H water molecule. A minuscule peak at 731 cm^−1^ was observed in the CeO_2_ spectrum but was not seen in the doped samples. As the doping concentration of Gd increased, the peak's intensity (absorbance) decreased, which may be due to the bending of Gd–O–Ce.^36,37^

### 3.5. UV-vis-NIR spectroscopy analysis

Regarding definitive optical strictures, ultraviolet-visible-near infrared (UV-vis-NIR) spectroscopy is the way to go. Depending on the shape and properties of the material under examination, the optical spectra obtained from UV-vis-NIR spectroscopy can exist in several modes, such as transmittance (*T*), reflectance (*R*), and absorption (*A*). The Tauc technique, which uses the absorption (*A*) and/or transmittance (*T*) spectra of UV-vis-NIR spectroscopy, can be used to estimate the band gap (*E*_g_) of a transparent sample. On the other hand, a non-transparent sample has low transparency, often approaching zero, and the *E*_g_ can be determined from the diffuse reflectance spectra (DRS). The current study used reflectance mode UV-vis-NIR spectroscopy on GDC ceramics at RT in the *λ* range of 200 to 2500 nm.

**Fig. 7:**
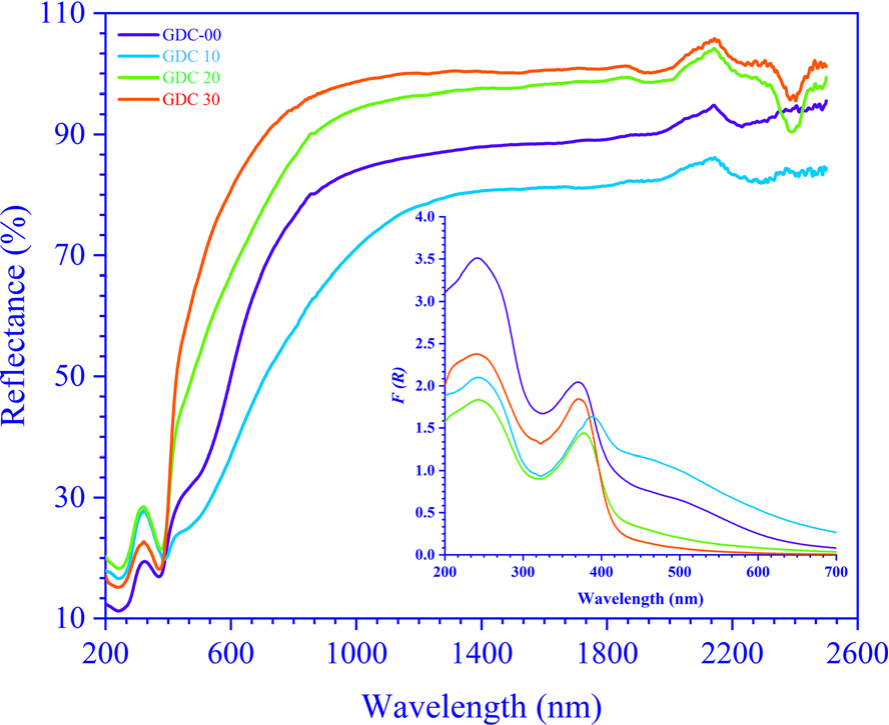
Reflectance spectra of GDC-00, -10, -20, and -30 ceramics (inset: *F*(*R*) spectra).

As a function of *λ*, Fig. 7 shows the *R* spectra, and 
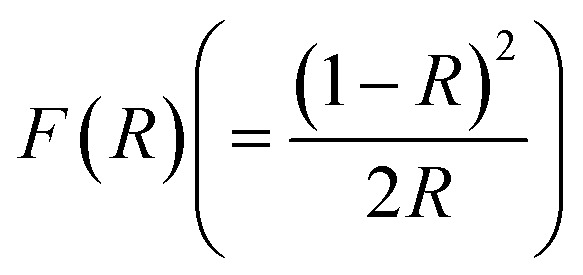
 GDC ceramics spectra were computed using the Kubelka–Munk (K–M) theory. The observed maximum *λ* shifts from ≃240 (11.23% *R*) to ≃370 (16.90% *R*) nm for GDC-00, ≃242 (16.58% *R*) to ≃388 (19.75% *R*) nm for GDC-10, ≃242 (18.24% *R*) to ≃377 (21.46% *R*) nm for GDC-20, and ≃239 (15.14% *R*) to ≃370 (18.16% *R*) nm for GDC-30, designate the establishment of CeO_2_ (GDC-00) and Gd-doped CeO_2_ (GDC-10, -20, and -30) due to the electron transition from O-2p to Ce-4f.^38^

**Fig. 8:**
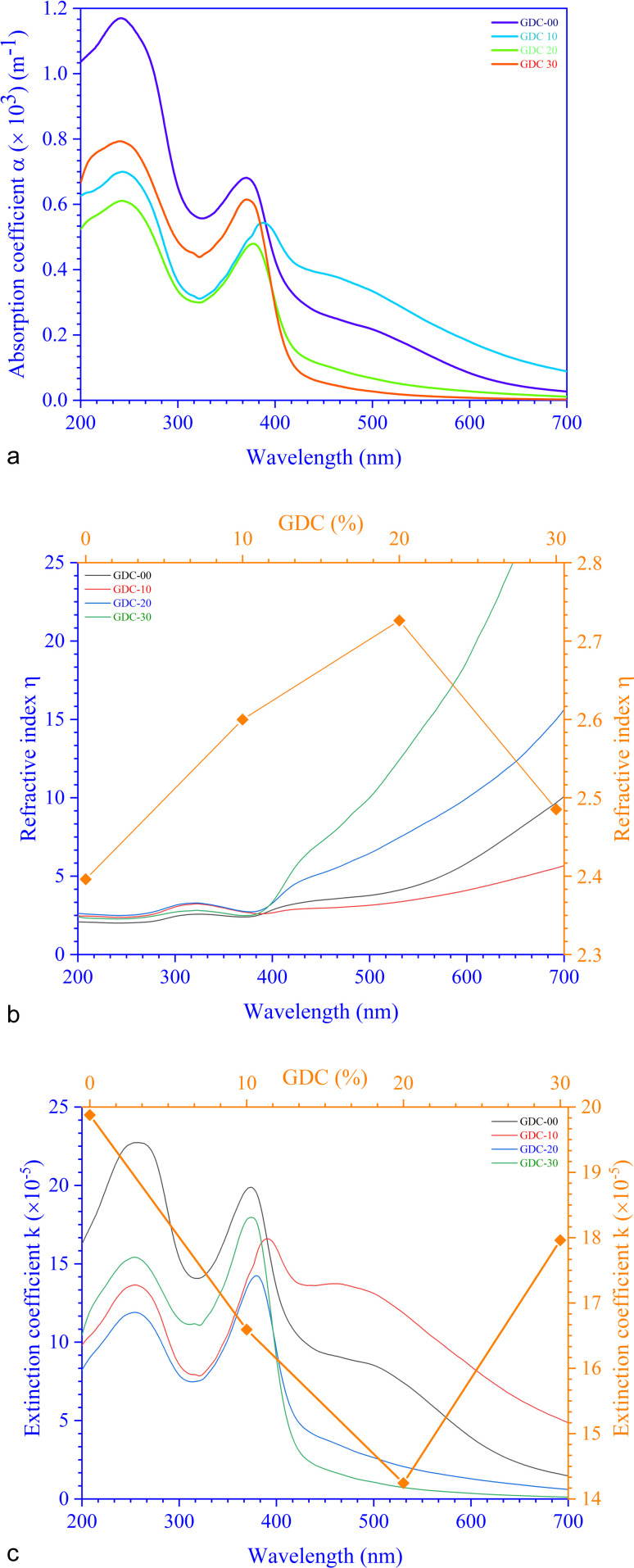
(a) Absorption coefficients, (b) refractive indexes, and (c) extinction coefficients of GDC ceramics.

For GDC ceramics, Fig. 8 shows how the optical parameters *α* (=
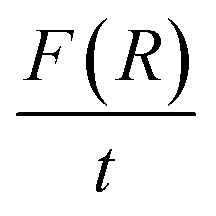
, where *t* (≃3 mm) is the thickness of the GDC pellets), *η* (
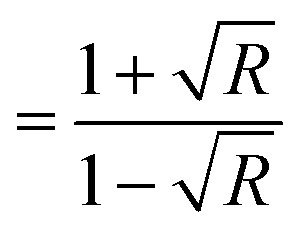
), and *k* (
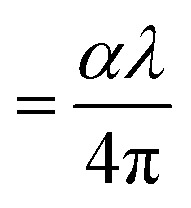
) vary concerning *λ*. The spectra of the absorption coefficient (*α*) in Fig. 8(a) revealed two distinct peaks at ≃240 and ≃370 nm for GDC-00, ≃242 and ≃388 nm for GDC-10, ≃242 and ≃377 nm for GDC-20, and ≃239 and ≃370 nm for GDC-30; these peaks can be interpreted as the result of the transition from the O-2p state of the valence band (VB) to the Ce-5d state of the conduction band (CB), and back again to the O-2p state of the VB to the Ce-4f state of the CB, respectively. A key feature of composite materials is the *R*-dependent *η*, which is strongly related to the electronic polarizability of ions and the local field within the material; Fig. 8(b) shows that *η* was found to be ≃2.3961 at a *λ*_c_ (≃370 nm) for GDC-00, ≃2.5998 at a *λ*_c_ (≃388 nm) for GDC-10, ≃2.7261 at a *λ*_c_ (≃378 nm) for GDC-20, and ≃2.4852 at a *λ*_c_ (≃370 nm) for GDC-30,^39^ and ties well with the empirical relation 
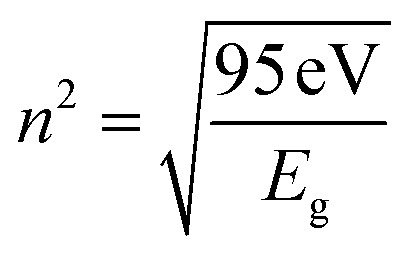
.^40^ As *λ* increases, *η* rises due to higher absorption at longer *λ*.^41^ The absorption of electromagnetic waves in semiconductors because of inelastic scattering events is reflected in the extinction coefficient (*k*), which is a measure of the fraction of light energy lost by scattering and/or absorption per unit distance of transit in a medium like ceramic. The value of *k*, which is directly proportional to *α*,^42^Fig. 8(c), was determined to be ≃19.8782 × 10^−5^ at a characteristic wavelength *λ*_c_ (≃374 nm) for GDC-00, ≃16.5930 × 10^−5^ at a *λ*_c_ (≃391 nm) for GDC-10, ≃14.2428 × 10^−5^ at a *λ*_c_ (≃379 nm) for GDC-20, and ≃17.9612 × 10^−5^ at a *λ*_c_ (≃374 nm) for GDC-30.

Combining the Tauc and K-M techniques, as shown in eqn (17), allows one to determine the optical *E*_g_ of GDC ceramics:^43^17
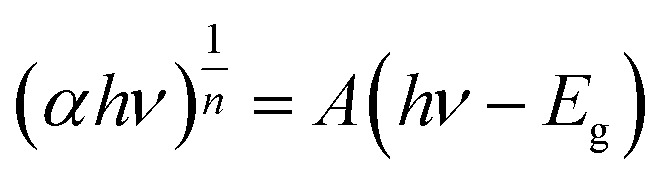


**Fig. 9:**
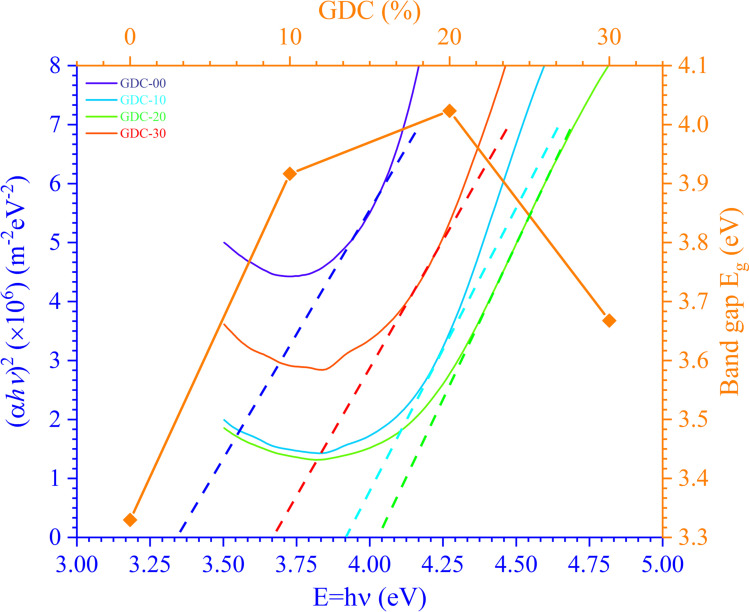
*E*_g_ plot for GDC ceramics.

The photon energy is denoted by *hν*, the transition probability is denoted by *n*, and *A* is a constant. Fig. 9 shows the plot of (*αhν*)^2^ against *hν*, which is used to find the optical *E*_g_ of GDC ceramics. The direct optical *E*_g_ value may be determined by extrapolating the straight section of the graphs at the values where (*αhν*)^2^ = 0; the results for the direct *E*_g_ for GDC ceramics are shown in Table 4.

**Table 4:** *E*_g_ values for GDC ceramics

System	GDC-00	GDC-10	GDC-20	GDC-30
*E*_g_ (eV)	3.3297	3.9166	4.0233	3.6676

**Fig. 10:**
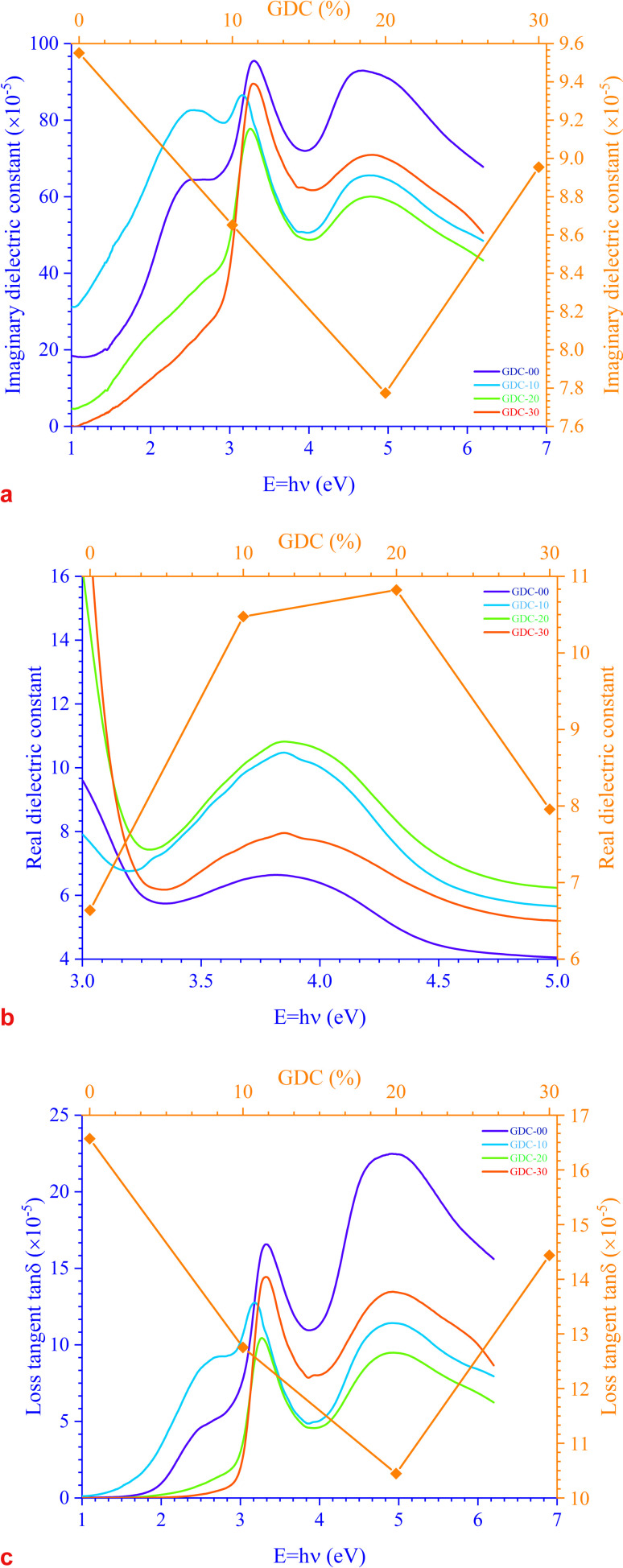
Variation in (a) IDC, (b) RDC, and (c) loss tangent as a function of energy for GDC ceramics.

There are two parts to the complex optical dielectric function: the imaginary (*ε*_*i*_) and the real (*ε*_r_) components; the former represents the absorption of energy from an electric field because of dipole motion, and the latter represents the capacity of materials to reduce the speed of light; *ε*_*i*_ and *ε*_r_ have unswerving relationships with the *η* and *k*. Fig. 10 shows the relationship between energy and the changes in the imaginary dielectric constant (IDC) (*ε*_*i*_)(=2*ηk*) (Fig. 10(a)), real dielectric constant (RDC) (*ε*_r_)(=*η*^2^ − *k*^2^) (Fig. 10(b)), and loss factor/loss tangent/dissipation factor 
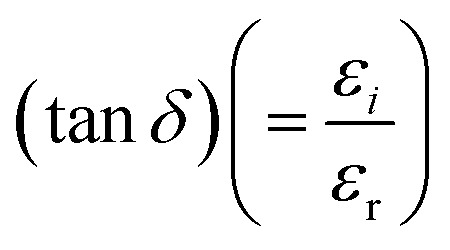
 (Fig. 10(c)) for GDC ceramics. The behavior of the plot is identical for both constants, *ε*_*i*_ and *ε*_r_. For GDC-00, the values of *ε*_*i*_, *ε*_r_, and tan *δ* are ≃95.5072 × 10^−5^, ≃6.6384, and ≃16.5694 × 10^−5^, respectively. For GDC-10, the values are ≃86.5184 × 10^−5^, ≃10.4754, and ≃12.7537 × 10^−5^. For GDC-20, the values are ≃77.7433 × 10^−5^, ≃10.8217, and ≃10.4463 × 10^−5^. Additionally, for GDC-30, the values are ≃89.5432 × 10^−5^, ≃7.9569, and ≃14.4378 × 10^−5^.^44^

**Fig. 11:**
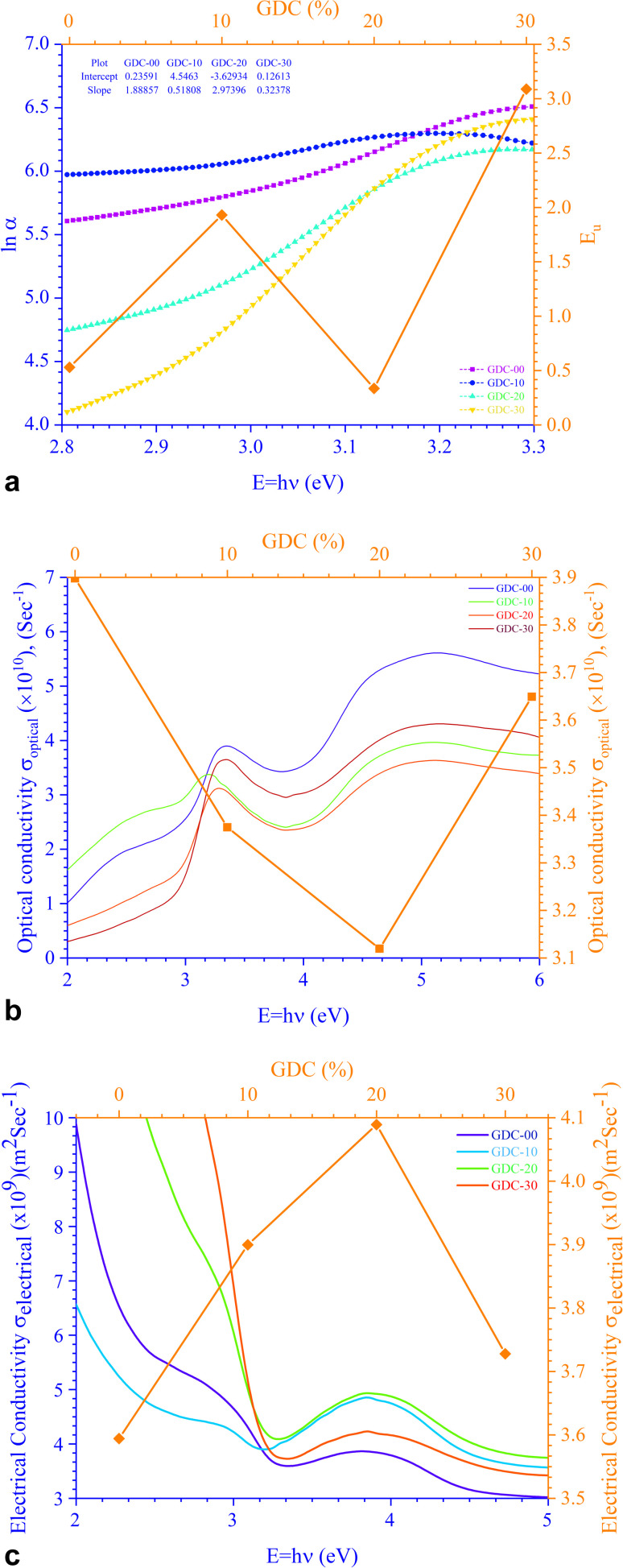
(a) Urbach energy, (b) optical conductivity, and (c) electrical conductivity for GDC ceramics.

The relationship between energy (*E*_g_) (=*hν*) and the changes in ln *α*, optical conductivity (*σ*_o_), and electrical conductivity (*σ*_e_) are shown in Fig. 11. An alteration in the optical state occurs when the VB tail becomes occupied, and the CB edge becomes unoccupied, as shown in eqn (18).^45^18
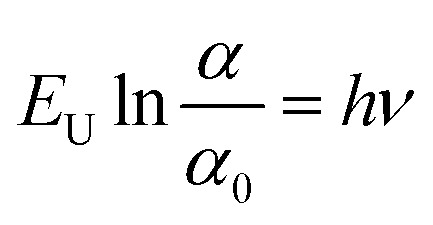


In this context, *α*_0_ represents an Urbach absorption coefficient (constant), and *E*_U_, which stands for the Urbach energy, determines the slope of the exponential edge and can be seen as the width of the tail of localized states in the forbidden energy gap. The thermal vibrations of the lattice form the basis of the *E*_U_. The values of the *E*_U_ and *α*_0_ attained from the plots of ln *α* against *hν* in Fig. 11(a) for GDC are as follows: ≃0.5295 eV & ≃1.2661 m^−1^ for GDC-00; ≃1.9302 eV & ≃94.2829 m^−1^ for GDC-10; ≃0.3363 eV & ≃0.0265 m^−1^ for GDC-20; and ≃3.0885 eV & ≃1.1344 m^−1^ for GDC-30.^19,46^ The high density of localized states inside the *E*_g_, as implied by the enormous value of the *E*_U_, revealed numerous structural flaws in the samples.^45^ The powerful probes, 
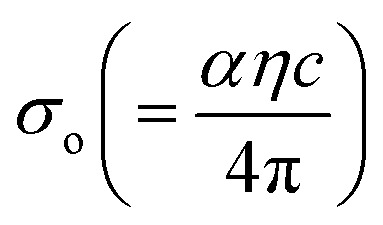
 and 
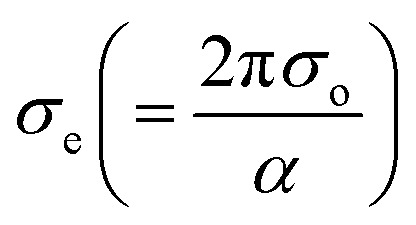
, represent the mobility of the charge carriers induced by the alternating the electric field of the passing electromagnetic waves^45^ and were employed in investigating the electrical properties of different materials. As shown in Fig. 11(b) and (c), the highest and lowest values of *σ*_0_ and *σ*_e_ for GDC-00, -10, -20, and -30 are ≃3.3423, ≃31 959, ≃3.2891, and ≃3.3514 eV for GDC-00, -10, -20, and -30, respectively.^47^

**Fig. 12:**
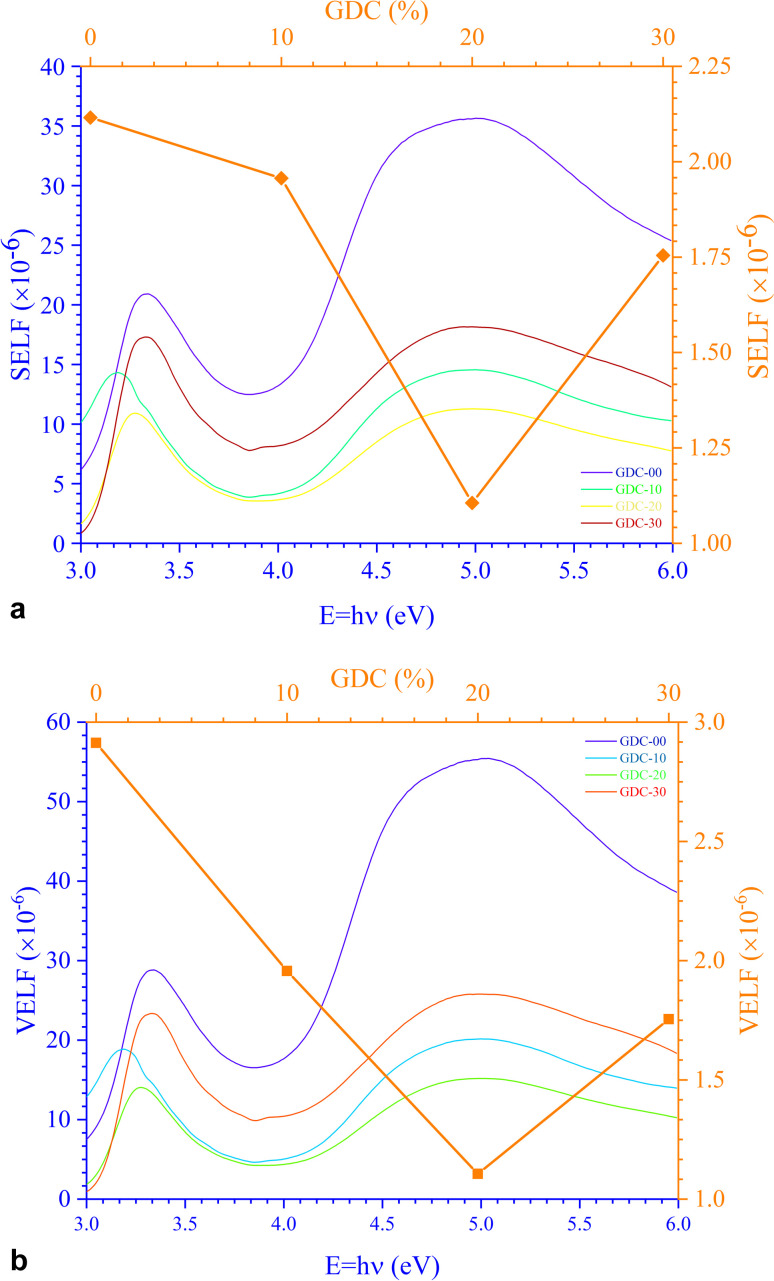
(a) Volume (VELF), and (b) surface (SELF) energy loss functions of GDC ceramics.

Fig. 12 portrays *ε*_*i*_ and *ε*_r_ constants, reliant volume 
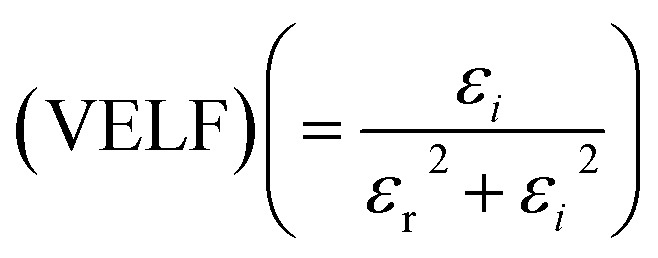
 (Fig. 12(a)) and surface 
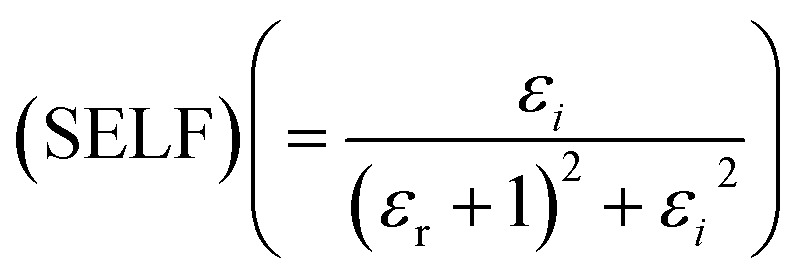
 (Fig. 12(b)) energy loss functions depicting the electron and optical transitions in GDC ceramics. VELF and SELF are used to determine the energy loss rates of electrons as they move across most of the surface. The idiosyncratic peaks of VELF and SELF for GDC were observed at ≃3.3423, ≃3.1877, ≃3.2718, and ≃3.3333 eV.^45^

### 3.6. Raman spectroscopy

**Fig. 13:**
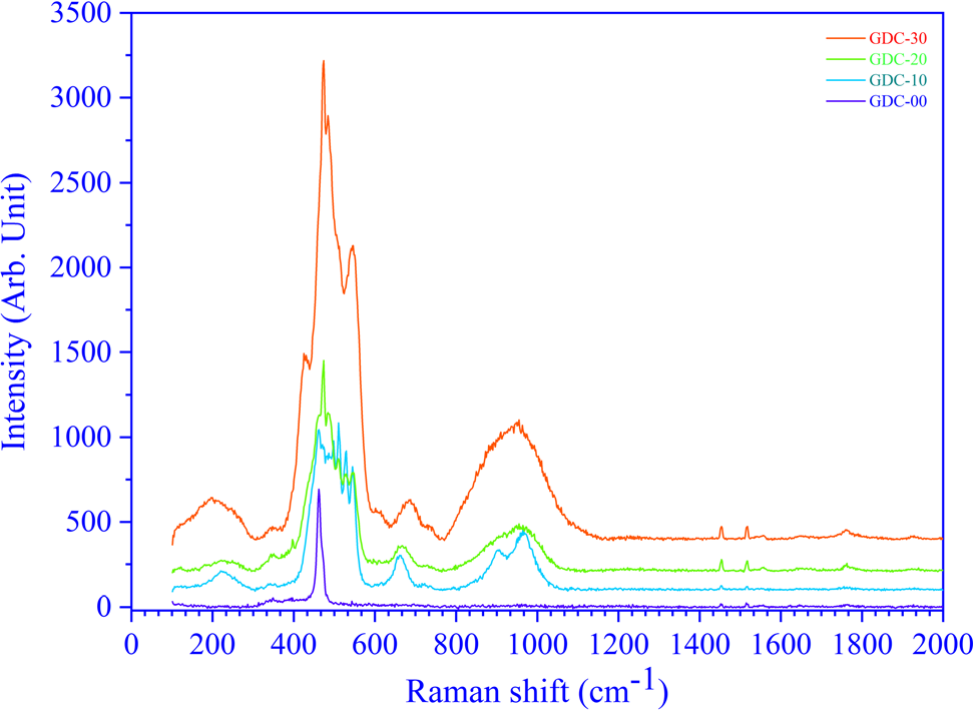
Raman spectra of GDC ceramics.

CeO_2_ exhibits a cubic fluorite structure with a space group *fm*3̄*m* (225), which has six optical phonon modes as per group theory; these modes correspond to the doubly degenerate transverse optical (TO) mode around 250 cm^−1^, the triply degenerate Raman-active mode (F_2g_) around 465 cm^−1^ and the non-degenerate longitudinal optical (LO) mode around 600 cm^−1^. TO and LO are infrared active modes. Fig. 13 shows the RT Raman spectra of GDC ceramics. The un-doped ceria (GDC-00) indicates only one sharp peak at 460 cm^−1^. The peak at 460 cm^−1^ is assigned to the F_2g_ symmetric mode of stretching vibrations of O atoms around the Ce atoms (Ce–O bond).^37^ The F_2g_ mode is susceptible to any changes in the metal and oxide bond length or the generation of oxygen vacancies. However, GDC-20 shows one sharp peak around 460 cm^−1^ and two broad peaks around 560 and 600 cm^−1^, with a smaller peak at 250 cm^−1^. The dopant Gd reduces the intensity and increases the width of the F_2g_ peak. However, the peaks at ≃250 cm^−1^, ≃560 cm^−1^, and ≃604 cm^−1^ are due to the substitution of Ce by Gd in the ceria fluorite structure.^37^ This substitution generates oxygen vacancies within the fluorite structure. The broad Raman band at ≃560 cm^−1^ appears because of the oxygen atom vibrating between Gd^3+^ ions and Ce^4+^ ions near oxygen vacancy defects. The Raman shift at ≃604 cm^−1^ originates from the vibration of oxygen atoms between Ce^4+^ and Gd^3+^ ions in the absence of oxygen defects.^37^

## 4. Conclusions

Pure and Gd-doped ceria have been synthesized *via* sol–gel combustion; evidence of both pure CeO_2_ and systems doped with Gd were supplied by the strong X-ray diffraction peaks that correspond to the reflection planes (1 1 1), (2 2 0), (3 1 1), (2 2 2), (4 0 0), (3 3 1), (4 2 0), and (4 2 2). The produced GDC ceramic samples exhibited high crystallinity as seen by their low FWHM value and marked XRD peak intensity. Crystallographically, the synthesized GDC ceramics belong to the *fm*3̄*m* (225) space group and feature a cubic architecture with a mono-phase. The computed *d*-interplanar spacings correlate well with the mentioned values; the SFs of GDC ceramics were determined. The *C*_*i*_ of each XRD peak was used to gauge the *σ* of crystallites along a crystal plane (*h k l*). The computed *d*_*h k l*_ indicates that *d*_1 1 1_ is dominant and *h*_1 1 1_ is the lowest, indicating the importance of the GDC's (1 1 1) plane. Results from various methods for determining the GDC ceramic's microstructural parameters, including N–R, Scherrer, S–W, Monshi, W–S, W–H, SSP, and H–W, are consistent. The densities of GDC ceramics were determined using a pycnometer. As a result of Ce–O and Gd–O–Ce bonds, GDC exhibited broad peaks in the RT FTIR spectra at 1025, 897, 1131, and 952 cm^−1^. The UV-vis-NIR spectrophotometer recorded the RT reflectance (R) spectra of GDC ceramics. The spectra of *α* suggest that GDC shows two distinct peaks, one at the O-2p state of the VB and the other at the Ce-5d state of the CB. *R*-dependent refractive index (*η*), *α*-dependent extinction coefficient (*k*), and optical band gap (*E*_g_) for the GDC-00, -10, -20, and -30 ceramic samples have been extracted. For GDC samples, the *ε*_*i*_, *ε*_r_, and tan *δ* were computed close to *λ*_c_. The results of the *E*_U_ and *α*_0_ calculations for the GDC ceramics are presented. *σ*_o_ and *σ*_e_ maxima and minima for GDC ceramics have been deduced. VELF and SELF were used to calculate the energy loss rates of electrons over the surface. Various vibrational modes in GDC ceramics were detected by Raman spectroscopy.

## Data availability

Data sharing does not smear this article, as no datasets were created or analyzed during the current study.

## Author contributions

“All writers contributed to the study conception and design. Material preparation, data gathering, and analysis were accomplished by S. D. Dhruv, Jayant Kolte, Pankaj Solanki, Milind P. Deshpande, Vanaraj Solanki, Jiten Tailor, Naveen Agrawal, V. A. Patel, J. H. Markna, Bharat Kataria, and D. K. Dhruv. D. K. Dhruv wrote the first draft of the manuscript, and all authors commented on previous versions. All authors read and permitted the final manuscript.”

## Conflicts of interest

“The authors have no related financial or non-financial interests to disclose.”

## Supplementary Material
